# Targeting of heme oxygenase-1 attenuates the negative impact of Ikaros isoform 6 in adult BCR-ABL1-positive B-ALL

**DOI:** 10.18632/oncotarget.10725

**Published:** 2016-07-20

**Authors:** Xiaojing Lin, Xingli Zou, Ziming Wang, Qin Fang, Shuya Chen, Jun Huang, Nana Zhe, Meisheng Yu, Yaming Zhang, Jishi Wang

**Affiliations:** ^1^ Clinical Medicine, Guizhou Medical University, Guiyang 550004, China; ^2^ Department of Hematology, The Affiliated Hospital of Guizhou Medical University, Guiyang 550004, China; ^3^ Department of Hematology, Guizhou Provincial Laboratory of Hematopoietic Stem Cell Transplantation Center, Guiyang 550004, China; ^4^ Department of Pharmacy, The Affiliated Baiyun Hospital of Guizhou Medical University, Guiyang 550004, China; ^5^ Department of Hematology, The Affiliated Hospital of North Sichuan Medical College, Nanchong 637000, China

**Keywords:** acute lymphoblastic leukemia, BCR-ABL1-positive, Heme oxygenase-1, IKZF1, STAT5

## Abstract

The correlation between Heme oxygenase-1 (HO-1) and dominant-negative Ikaros isoform 6 (IK6) is unclear. Firstly, we detected that IK6 existed in 20 of 42 (47.6%) adult BCR-ABL1-positive B-lineage acute lymphoblastic leukemia (BCR-ABL1-positive B-ALL) by using reverse transcribed polymerase chain reaction (PCR) and nucleotide sequencing. IK6-positive patients had an unfavorable outcome compared with IK6-negative ones. Further study showed that the level of HO-1 expression was higher in IK6-positive patients’ samples than that in IK6-negative ones. And there was a strong correlation between the expression of IK6 and HO-1. The growth of primary CD34^+^ leukemic cells derived from our IK6-positive patients’ pool was prohibited by silencing HO-1, further promoting their apoptosis. Furthermore, primary CD34^+^ leukemic cells derived from IK6-positive patients shown poor responses to imatinib in comparison with wild-type (IK1) patients, suggesting that the expression of IK6 resisted to imatinib in adult BCR-ABL1-positive B-ALL. Importantly, inhibition of HO-1 also increased their sensitivity to tyrosine kinase inhibitors (TKIs). Finally, we found that IK6 activated downstream STAT5, and HO-1 was one of the downstream target genes of STAT5. In conclusion, HO-1 is an essential survival factor in BCR-ABL1-positive B-ALL with IK6, and targeting HO-1 can attenuate the negative impact of IK6.

## INTRODUCTION

Acute lymphoblastic leukemia (ALL) is a heterogeneous disease with multiple genetic aberrations which are prognostically relevant [1,2]. The BCR gene, chromosome region 22q11, translocating into ABL1 gene, chromosome region 9q34, results in the BCR-ABL1 fusion transcript (t(9;22) (q34;q11)). And it is a highly unfavorable abnormality among genetic aberrations [1,3,4].

Besides, IKZF1 deletions have been also considered as indicators of poor prognosis in B-ALL [5–9]. The IKZF1 gene encoding Ikaros, a DNA-binding zinc finger protein, involves in B-cell proliferation and differentiation [10,11]. IK1, known as IKZF1 wild type, contains 7 exons (exons 2 - 8). It totally encodes 6 Cys-2 to His-2 zinc fingers, 4 at its N-terminus (F1, F2, F3, and F4) and 2 at its C-terminus (F5 and F6) [12,13]. Ikaros isoforms can also take a function of DNA-binding when there are at least three DNA-binding zinc fingers. However, the lack of two or more zinc-finger domains will impair the function of Ikaros proteins in a dominant-negative manner [14]. Mounting studies have revealed that the frequency of IKZF1 deletions is high (70%) in BCR-ABL1-positive B-ALL [9,15,16], and the most common one is IK6 which affects the DNA-binding domain in exons 4-7. IK6 exerts a dominant-negative effect over the unaffected allele, which results into a loss of the tumor suppressor [17,18]. Moreover, the expression of IK6 usually predicts an adverse clinical outcome [8] and the insensitivity to TKIs [9,13]. Lots of experimental data are available for pediatric B-ALL but for adults. Despite limited data, it appears to be true for adults, too [19,20]. The BCR-ABL1 fusion transcript itself is a strong adverse risk factor, and the existence of extra IK6 seems to exacerbate poor prognosis and risks of relapse. Thus, it is even wiser to figure out whether there are alternative therapies for patients with the expression of IK6.

HO-1 is a stress-related cytoprotective molecule. In our previous works, we found that it inhibited the apoptosis and promoted the proliferation of leukemic cells, being associated with tumorigenesis and drug resistance [21–23]. Recent studies show that HO-1 is considered as a survival molecule to play an important role in BCR-ABL1-positive leukemic cells, and HO-1-targeting drugs are able to synergize with TKIs to inhibit growth and promote apoptosis of leukemic cells [24–26]. Therefore, HO-1 is an essential survival factor and potential target in BCR-ABL1-positive leukemias.

Although IKZF1 deletions are under the spotlight in pediatric BCR-ABL1-positive B-ALL, there are few studies on adults. Therefore we chose adult B-ALL as the research object. As noted, IK6 is highly associated with the BCR-ABL1 fusion oncogene [13], meanwhile HO-1 is identified as a novel BCR-ABL1-driven survival molecule [24,25]. It is worthwhile to make a connection between HO-1 and IK6. There is assuming a correlation, whether targeting HO-1 can attenuate the negative impact of IK6 in adult BCR-ABL1-positive B-ALL, and how. To address these issues, we analyzed the expression of HO-1 in 42 adult BCR-ABL1-positive B-ALL. We further did studies on cell proliferation, apoptosis and cell cycle by increasing or decreasing the expression of HO-1 in primary CD34^+^ leukemic cells from our patients’ pool. After the inhibition of HO-1, the experiment on the sensitivity of primary leukemic cells to TKIs was carried out. As previously demonstrated in murine B lymphocytes [27], IKZF1 deletions may synergize with STAT5 activation in the presence of BCR-ABL1 [12,27]. Therefore, it will be important to figure out the relationship among IK6, STAT5 and our target gene HO-1. Based on this, we studied the molecular pathways involved in BCR-ABL1-positive B-ALL with IK6.

## RESULTS

### The IK6 is highly expressed in BCR-ABL1-positive B-ALL

**Figure 1:**
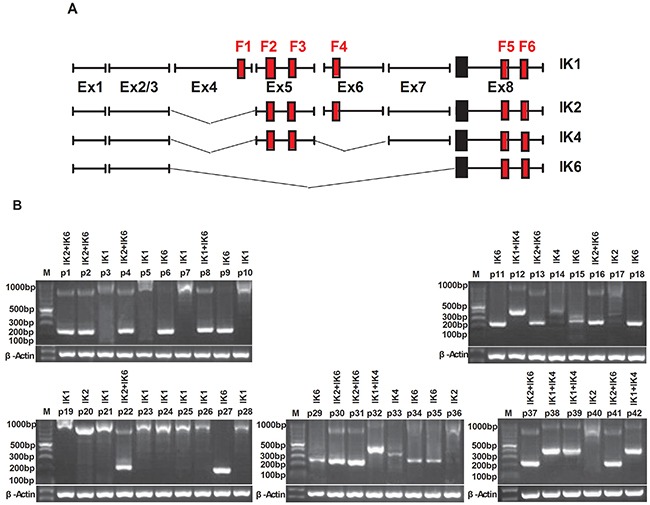

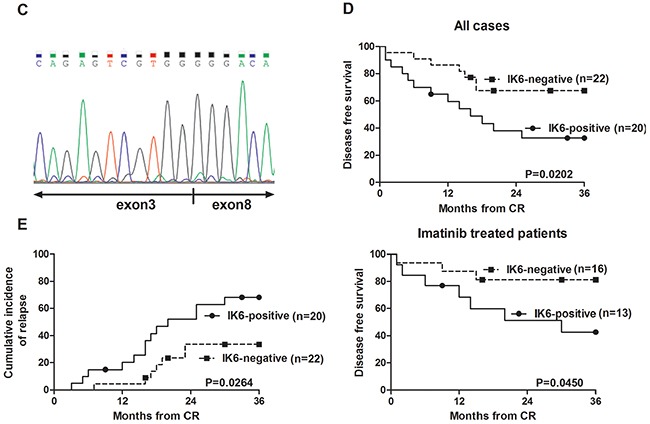
IK6 is highly expressed in adult BCR-ABL1-positive B-ALL patients **A.** Schematic diagram of the full-length IKZF1 cDNA and the different isoforms detected in our samples. F: N-terminal zinc-fingers show DNA-binding activity, and C-terminal zinc fingers mediate dimerization of the protein. Ex indicates exon. **B.** Expressions of the different IKZF1 isoforms in 42 BCR-ABL1-positive B-ALLsamples were determined by RT-PCR. IK1(945bp), IK2(884bp), IK4 (458bp), IK6(255bp). **C.** Sequencing analysis of IK6 isoform in which exon 3 is juxtaposed with exon8. **D.** Kaplan-meier estimation of DFS in IK6-positive and -negative group. **E.** Cumulative incidence of relapse curves by IKZF1 status in our samples, with 3-year estimates. CR1, first complete remission.

We analyzed the expression of different IKZF1 isoforms in 42 adult BCR-ABL1-positive B-ALL by using RT-PCR and nucleotide sequencing. 4 subtypes of IKZF1, IK1, IK2, IK4 and IK6 (Figure 1A), were detected in our study. Results shown that patients containing either IK6 or IK4 accounted for 64.2% (27 of 42). IK4 was only expressed in 7 patients. There were 9 patients exclusively expressed IK6 in the 20 patients (47.6%) who expressed IK6, while the remaining 11 patients were accompanied with the expression of IK1 or IK2. Additionally, another 11 patients exclusively expressed IK1(26.2%), and the left 4 patients only present IK2 (Figure 1B, Table 1). Data from gene sequencing also validated the structure of IK6 as described above (Figure 1C). Usually, BCR-ABL1 fusion gene predominantly transcribes into either p210 or p190 oncoprotein, but our data further demonstrated the existence of IK6 did not limit to a specific BCR-ABL1 subtype (Table 2). Moreover, experimental results shown that IK6 expression was significantly correlated with higher white blood cell (WBC) count, minimal residual disease (MRD) and poor early clinical response, but neither gender nor age (Table 2). We also found that IK6-positive patients had a lower disease free survival (DFS) compared with IK6-negative ones, even when treated with imatinib (Figure 1D), and with a higher incidence of relapse (Figure 1E). These findings confirm that IK6 is highly expressed in adult BCR-ABL1-positive B-ALL, and it is closely associated with a poor outcome.

**Table 1:** Expression of different IKZF1 isoforms in BCR-ABL1-positive B-ALL(n=42)

Expression of different IKZF1 isoforms	Positive
IK1	17(40.5%)
Pts expessing only IK1	11
Pts co-expessing IK6	1
Pts co-expessing IK4	5
IK2	14(33.3%)
Pts expressing only IK2	4
Pts co-expressing IK6	10
IK4	7(16.7%)
Pts expressing only IK4	2
Pts co-expressing IK1	5
IK6	20(47.6%)
Pts expressing only IK6	9
Pts co-expressing IK1	1
Pts co-expressing IK2	10

Abbreviation: Pts=patients.

**Table 2:** Patients’ characteristics

Clinical parameter	IK6-positive		IK6-negative		P value
	(n=20)		(n=22)		
	N	%	N	%	
**Gender**					
Male	11	55	13	59.1	0.789
Female	9	45	9	40.9	
**Age(years)**					
18-39	10	50	12	54.5	0.918
40-64	6	30	7	31.8	
≥65	4	20	3	13.6	
**White blood cells count (cells×10^9^/L)**					
<30	4	20	15	68.2	**0.005**
30-100	6	30	4	18.2	
>100	10	50	3	13.7	
**aEarly clinical response**					
Yes	6	30	15	68.2	**0.013**
No	14	70	7	31.8	
**BCR-ABL1**					
p190	11	55	14	63.6	0.569
p210	9	45	8	36.4	
**Imatinib exposure**					
Yes	13	65	16	72.7	
No	7	35	6	27.3	
**bHSCT**					
Yes	4	20	7	31.8	
No	16	80	15	68.2	
**cMRD risk group**					
Standard risk	4	20	12	54.5	**0.036**
Intermediate risk	9	45	8	36.4	
High risk	7	35	2	9.1	

a **Early clinical response** was defined as≤5% leukemic blast cells in the bone marrow at day 21 (depending on national induction protocols).

b **HSCT**, hematopoietic stem cell transplantation.

c **Minimal residual disease (MRD)**, standard risk: negative after induction therapy on treatment day 33 and consolidation therapy on treatment day 78, high risk: still positive at an MRD level of ≥10^3^ after consolidation therapy on treatment day 78, intermediate risk: all others.

### The expression of HO-1 is strongly correlated with IK6

**Figure 2:**
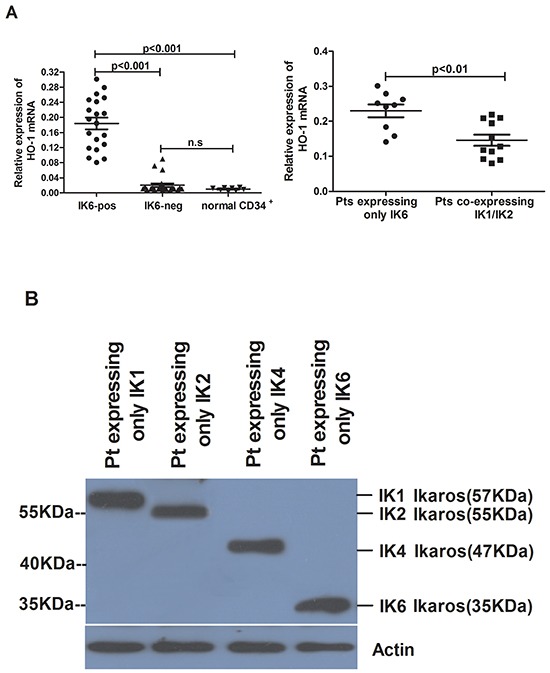

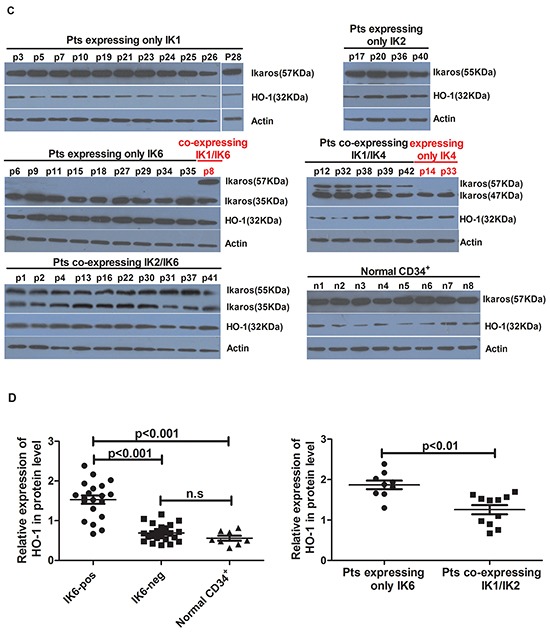

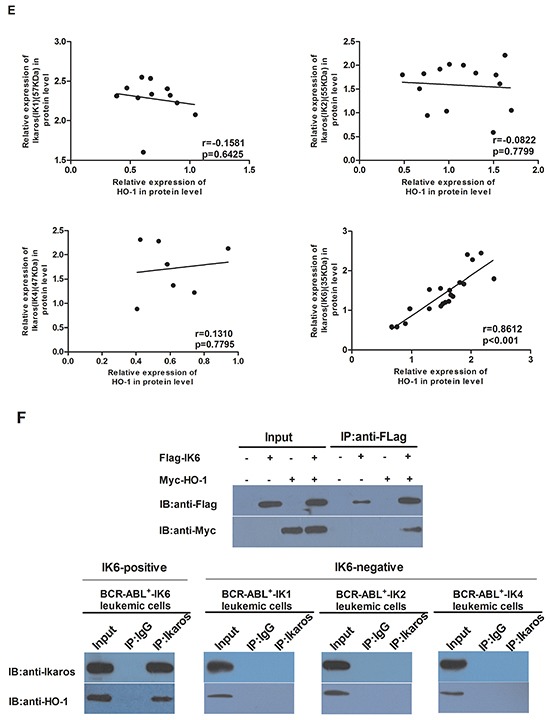
The expression of HO-1 is strongly correlated with IK6 **A.** Differential expression of HO-1 mRNA in adult BCR-ABL1-positive B-ALL patients and normal CD34^+^ cells were determined by qRT-PCR. **B.** Western blot analysis of Ikaros expression in IK6-positive and –negative patients. **C, D.** Western blot analysis of HO-1 expression at protein level in adult BCR-ABL1-positive B-ALL patients and normal CD34^+^ cells. **E.** Scatterplot representation of the correlation between the expression of HO-1 and Ikaros in patient samples. **F.** Co-immunoprecipitation assay to verify the interaction of Flag-IK6 with Myc-HO-1 in SupB15 cells (upper panel) and the endogenous interaction of Ikaros with HO-1 in primary leukemic cells (lower panel). (Pts=patients).

We further analyzed the expression of HO-1 in patients’ samples by using qRT-PCR and western blot. Compared to normal CD34^+^ cells, the level of HO-1 expression was higher in IK6-positive patients’ samples, but there was no obvious increase in IK6-negative samples. Furthermore, the expression of HO-1 in IK6-positive patients was 4 folds more than that in IK6-negative ones (Figure 2A). Then the expressions of Ikaros and HO-1 were detected by western blot. Primary leukemic cells expressed a 57kDa immunoreactive protein corresponding in size to IK1, a 55kDa immunoreactive protein corresponding in size to IK2 and a 47kDa immunoreactive protein corresponding in size to IK4. In contrast, we confirmed the presence of a smaller immunoreactive protein band of approximately 35 kDa, which corresponded in size to IK6 (Figure 2B). HO-1 were also higher at the protein level in IK6-positive samples. Significantly, 15 of 20 (75%) IK6-positive patients were detected the overexpression of protein by western blot study (Figure 2C, 2D). Because of the status of IKZF1 isoforms, there was a strongly statistically significant difference in HO-1 expression of the 20 IK6-positive patients. Specifically, 9 patients who expressed only IK6 expressed more HO-1 than those who co-expressed with another IKZF1 isoform (IK1 or IK2) at both mRNA and protein level (Figure 2A, 2D). In addition, HO-1 had no correlation with Ikaros(57KDa)(IK1), Ikaros(55KDa)(IK2) and Ikaros(47KDa)(IK4), but was positively correlated with Ikaros (35KDa) (IK6) (Figure 2E). Further, co-immunoprecipitation assay in SupB15 cells showed that Flag-tagged IK6 interacted with Myc-tagged HO-1. We further showed that endogenous Ikaros was co-immunoprecipitated with HO-1 in IK6-positive patients, but not in IK6-negative samples (Figure 2F). Hence, these results show that the expression of HO-1 is strongly correlated with IK6.

### Inhibiting HO-1 reduces the proliferation and induces the apoptosis of BCR-ABL1^+^-IK6 leukemic cells

10 adult BCR-ABL1-positive B-ALL patients, 3 (marked as pI, pII, pIII) only expressed IK1(BCR-ABL1^+^-IK1), 3 (pIV, pV, pVI) only expressed IK6 (BCR-ABL1^+^-IK6), 2 only expressed IK2 (BCR-ABL1^+^-IK2) and 2 only expressed IK4 (BCR-ABL1^+^-IK4) were randomly selected, and their primary CD34^+^ leukemic cells (BCR-ABL1^+^-IK6 leukemic cells and IK6-negative leukemic cells, including BCR-ABL1^+^-IK1 leukemic cells, BCR-ABL1^+^-IK2 leukemic cells and BCR-ABL1^+^-IK4 leukemic cells) at diagnosis were used for transfection.

**Figure 3:**
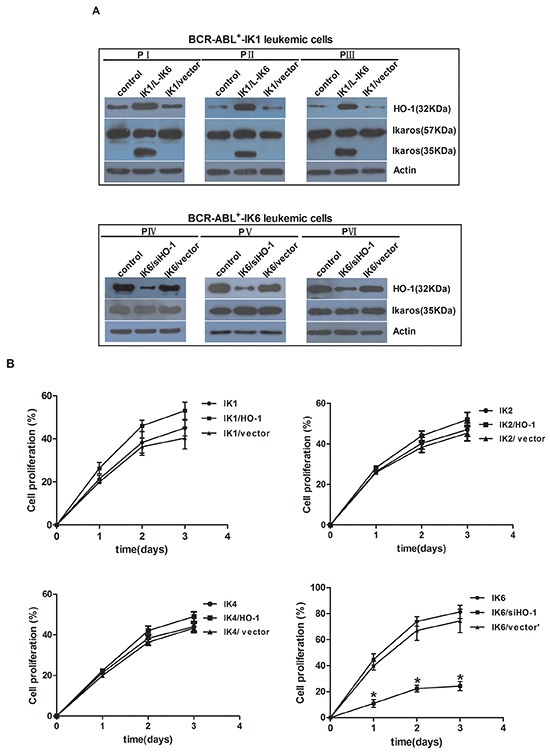

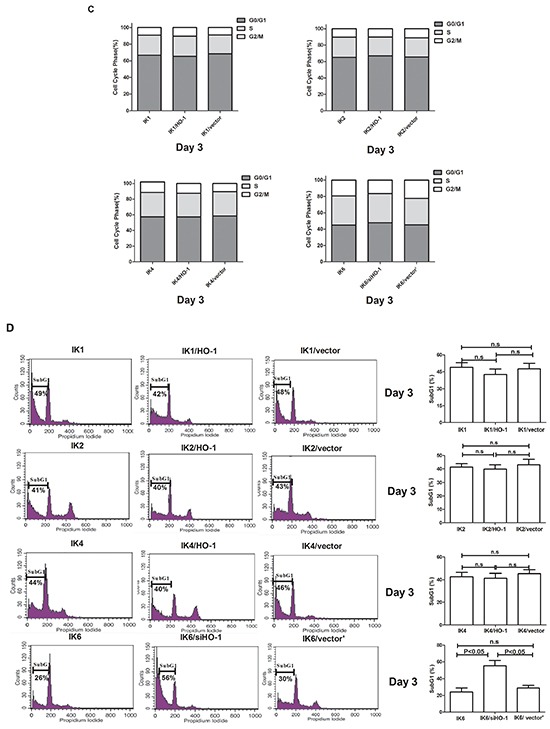

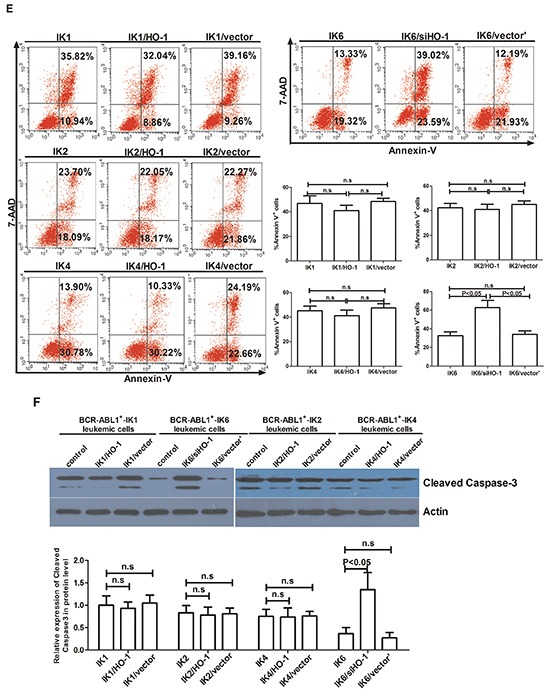
Inhibition of HO-1 reduces proliferation and induces apoptosis in IK6^+^ cells **A.** Expressions of HO-1 and Ikaros were detected by western blot after cells transfection. **B.** Proliferation kinetics of primary CD34^+^ leukemic cells as determined by trypan blue exclusion. Values are expressed as means ± sd, *P < 0.05. After transfection for 72h, cells which were cultured in the absence of mix 3-cytokine were harvested at the third day. **C, D.** cell cycle (Propidium iodide (PI)). **E.** apoptosis (7-AAD and Annexin V) were performed. **F.** Whole-cell lysates were subjected to western blot to determine Cleaved Caspase 3. IK1, IK2, IK4 and IK6 represents BCR-ABL1^+^-IK1 leukemic cells, BCR-ABL1^+^-IK2 leukemic cells, BCR-ABL1^+^-IK4 leukemic cells and BCR-ABL1^+^-IK6 leukemic cells, respectively.

Firstly, we used BCR-ABL1^+^-IK1 leukemic cells and BCR-ABL1^+^-IK6 leukemic cells as tools to investigate whether the expression of HO-1 was activated by IK6. The expression of HO-1 increased significantly in IK6-transduced BCR-ABL1^+^-IK1 leukemic cells (IK1/L-IK6). However, the expression of Ikaros (35KDa) was not significantly changed after silencing HO-1 in BCR-ABL1^+^-IK6 leukemic cells (IK6/siHO-1) (Figure 3A). This demonstrated that HO-1 was activated by IK6.

Further data showed that the proliferation of HO-1-transduced IK6-negative leukemic cells(IK1/HO-1, IK2/HO-1and IK4/HO-1) was slightly increased compared to control. In contrast, the rapid proliferation of BCR-ABL1^+^-IK6 leukemic cells was most inhibited by silencing HO-1 (Figure 3B). To study the impact of HO-1 on cell cycle and apoptosis, transfected cells were analyzed for DNA content after culturing for 3 days in the absence of mix 3-cytokine. No matter how to increase or decrease HO-1, cell cycle was only marginally changed in the four groups (Figure 3C). The sub G1 fraction (which represents the apoptosis) of BCR-ABL1^+^-IK6 leukemic cells was increased from 24.2%±4.9% to 55.3%±6.4% by silencing HO-1, whereas IK6-negative leukemic cells almost unchanged (BCR-ABL1^+^-IK1 49%±3.9% VS 42%±4.7%, BCR-ABL1^+^-IK2 41.3±2.6% VS 39.8±4.2%, BCR-ABL1^+^-IK4 42.6±3.7% VS 41.2±4.3%, respectively, Day 3) by increasing the expression of HO-1 (Figure 3D). Similar results were obtained when cells were stained by AnnexinV. The number of Annexin V-positive cells was significantly increased in IK6/siHO-1group (Figure 3E). In consistent with the analysis of DNA content, the decrease of Cleaved Caspase 3 was not statistically significant in HO-1-transduced cells compared to that in IK6-negative leukemic cells. However, silencing HO-1 led to a marked increase in Cleaved Caspase 3 in BCR-ABL1^+^-IK6 leukemic cells (Figure 3F). These results indicate that the inhibition of HO-1 reduces the proliferation and induces the apoptosis of BCR-ABL1^+^-IK6 leukemic cells, but no effect on IK6-negative leukemic cells. In a word, IK6-dependent activation of HO-1 increases cell proliferation and markedly decreases apoptosis in BCR-ABL1-positive B-ALL.

### HO-1 inhibition increases the sensitivity of leukemic cells to TKIs in BCR-ABL1^+^-IK6 patients

**Figure 4:**
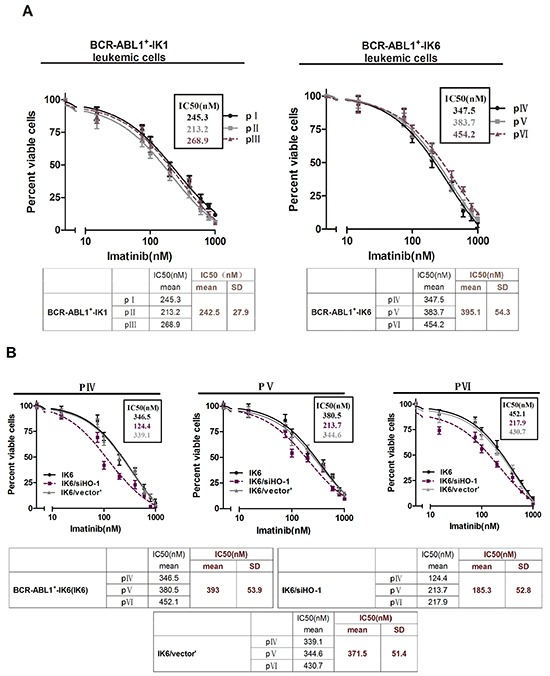

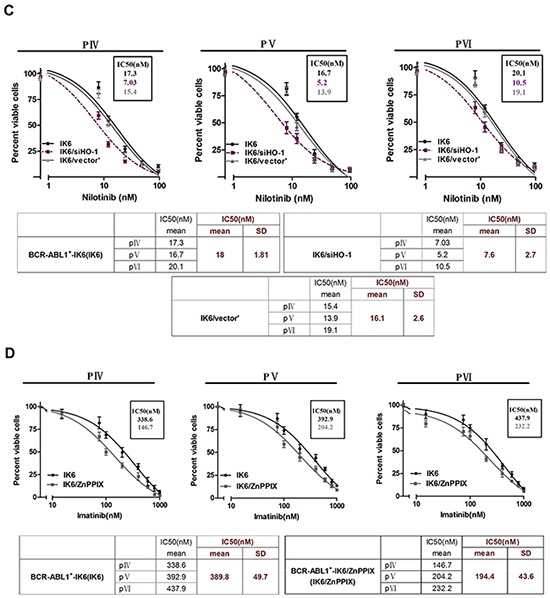
HO-1 inhibition increases the sensitivity of leukemic cells to TKIs in BCR-ABL1^+^-IK6 patients Cell viability was determined by MTT assay after 48 hours’ incubation. The IC50 mean and SD of primary leukemic cells from 3 patients in each group were shown in the tables. **A.** Primary CD34^+^ leukemic cells (4×10^4^ cells per well) were treated with different doses of imatinib (15nM,75nM,100nM, 200nM, 400nM, 600nM, 800nM, 1000nM) for 48 h. **B.** BCR-ABL1^+^-IK6 leukemic cells (IK6), IK6/siHO-1 and IK6/vector’ were incubated with different concentration of imatinib as indicated above. **C.** Equal numbers of IK6, IK6/siHO-1, IK6/vector’ were incubated with different concentration of nilotinib (8nM, 12nM, 24nM, 48nM, 96nM). **D.** Cells were treated with 10uM ZnPPIX and differrant concentration of imtinib as indicated above.

We also did experiments on patients’ leukemic cells to figure out the effects of inhibiting HO-1 on the sensitivity to TKIs, such as imatinib or nilotinib. Primary CD34^+^ leukemic cells directly from pIV, pV and pVI shown poor responses to imatinib in comparison with their counterparts (pI, pII and pIII) (IC50, mean±SD, 395.1±54.3nM VS 242.5±27.9nM) (Figure 4A), suggesting that the expression of IK6 probably resisted to imatinib. Importantly, silencing HO-1 in primary leukemic cells of pIV, pV and pVI sensitized their responses to imatinib with a significant decrease in the IC50 from 393±53.9nM to 185.3±52.8 nM (Figure 4B). Similar trends of IC50 (18±1.8nM to 7.6±2.7nM) also shown in nilotinib group, the most potent second-generation TKIs (Figure 4C). Inhibition of HO-1 by ZnppIX, a pharmacologic inhibitor of HO-1, in place of silencing HO-1 by siRNA lowered the IC50 to imatinib as well (Figure 4D). These results suggest that HO-1 inhibition in combination with TKIs may be a rational strategy for treating BCR-ABL1^+^-IK6 patients.

### IK6 activates STAT5 and HO-1 in BCR-ABL1^+^-IK6 leukemic cells

**Figure 5:**
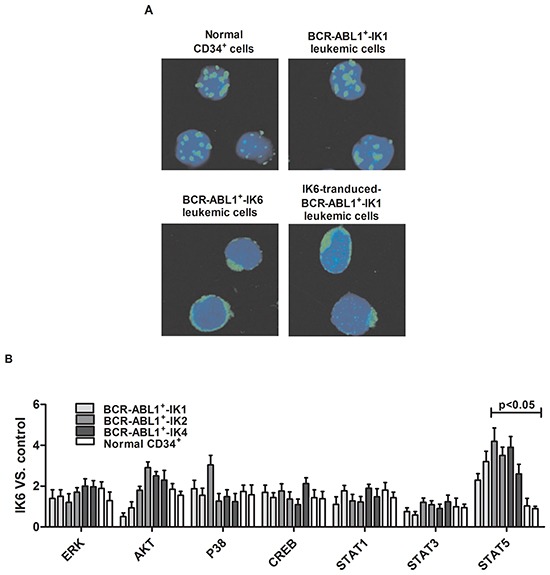

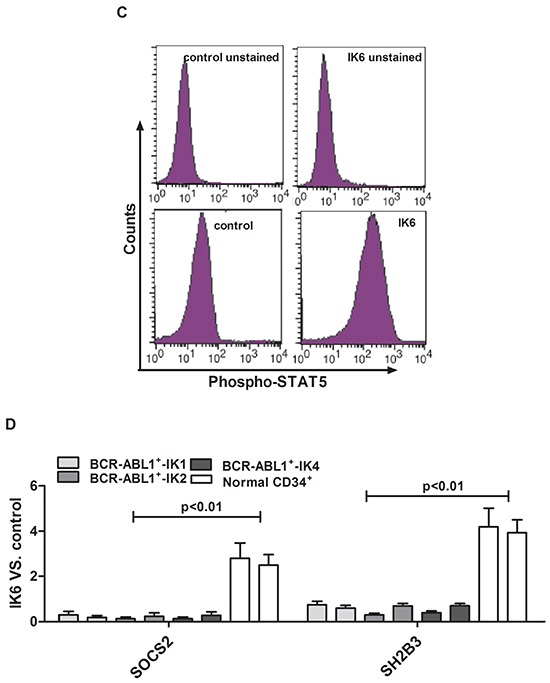

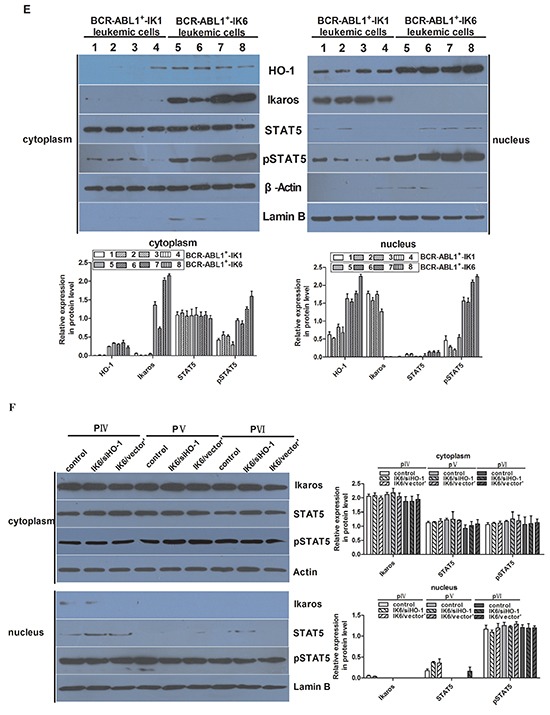

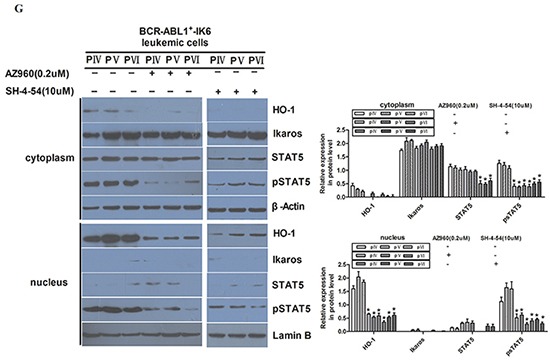
IK6 activates STAT5 and HO-1 in BCR-ABL1^+^-IK6 leukemic cells **A.** Confocal microscopy images of representative single primary CD34^+^ cells, either normal or BCR-ABL1-IK1, BCR-ABL1-IK6 and IK6-transduced BCR-ABL1^+^-IK1, and stained with DAPI (blue) and an antibody reactive with Ikaros (green). **B.** Phosphoprotein analysis of IK6- or control-transduced CD34^+^ cells shown individually for 2 BCR-ABL1^+^-IK1, 2 BCR-ABL1^+^-IK2, 2 BCR-ABL1^+^-IK4 and 2 normal BM samples. Values shown for each protein are the mean±SD of the ratios of the median fluorescent intensity values obtained for the IK6-compared with control-transduced cells in 3 replicate experiments. P values were generated by comparing the effects of IK6 on leukemic cells vs normal BM cells. **C.** Representative flow cytometry histograms taken from one of the patient experiments shown in panel B. **D.** Comparison of transcript differences in IK6- and control-transduced CD34^+^ cells for 6 IK6-negative and 2 normal BM samples. Values shown are the mean SD of the ratios of normalized transcript levels in IK6- as compared with control-transduced cells. **E.** Expressions of HO-1, Ikaros, STAT5, pSTAT5 in patients with either IK1 or IK6 detected by Western blot. 1, 2, 3, and 4 are samples of 4 randomly selected BCR-ABL1^+^-IK1 patients, 5, 6, 7 and 8 represent samples of 4 BCR-ABL1^+^-IK6 patients. **F.** Western blot analyzed the expressions of Ikaros, STAT5, pSTAT5 in different cells by silencing HO-1 expression. **G.** Expressions of HO-1, Ikaros, STAT5 and pSTAT5 were measured after treating with 0.2uM AZ906 or 10uM SH-4-54 for 48 hours.

We compared the subcellular localization of Ikaros protein in normal CD34^+^ cells and primary leukemic cells from patients by confocal microscopy. Results showed that BCR-ABL1^+^-IK6 leukemic cells caused Ikaros protein to be retained in the cytoplasm, in contrast to the normal localization of Ikaros in the nucleus observed in both normal CD34^+^ cells and BCR-ABL1^+^-IK1 leukemic cells. We also found that forced expression of IK6 in BCR-ABL1^+^-IK1 leukemic cells caused partial Ikaros protein to be retained in the cytoplasm (Figure 5A). These findings indicate an ability of IK6 to disrupt the nuclear transcription factor activity of Ikaros, which is consistent with previous reports [12,28].

Then we used flow cytometry and qRT-PCR to look for molecular pathways that are differentially activated in IK6-positive leukemic cells. We found that IK6-transduced BCR-ABL1^+^-IK1 or BCR-ABL1^+^-IK2 or BCR-ABL1^+^-IK4 leukemic cells contained consistently higher levels of pSTAT5 by comparison with those derived from either matched control-transduced cells or IK6-transduced normal CD34^+^ cells. Effects of IK6 on other signaling intermediaries in BCR-ABL1-positive B-ALL cells were more heterogeneous (Figure 5B, 5C). In light of these findings, we hypothesized that the expression of IK6 in BCR-ABL1-positive B-ALL leukemic cells may disrupt mechanisms regulating JAK2-STAT5 activity in them. Consistent with this postulate was the finding that IK6-expressing BCR-ABL-positive B-ALL leukemic cells contained decreased levels of SOCS2 and SH2B3 (LNK) transcripts, both of which encode important known negative regulators of JAK-STAT signaling [28]. Notably, none of these molecular effects were apparent in the IK6-transduced normal CD34^+^ cells (Figure 5D). These suggest that IK6 enhances STAT5 signaling in BCR-ABL1^+^-IK6 leukemic cell.

Next, our results of western blot were consistent with confocal examination. Our data also demonstrated that HO-1 was mainly expressed in the nucleus, too (Figure 5E). Secondly, the expression of IK6 was not only associated with the increase of pSTAT5 in both cytoplasm and nucleus, but also the increase of HO-1 in nucleus (Figure 5E). However, the expressions of Ikaros and pSTAT5 were not significantly changed after silencing HO-1 in BCR-ABL1^+^-IK6 leukemic cells (Figure 5F). Although AZ960 can block the phosphorylation of STAT5 [29,30], the expression of Ikaros protein was not significantly changed after treating BCR-ABL1^+^-IK6 leukemic cells with 0.2uM AZ960 for 48 hours. Contrarily, the expression of HO-1 was significantly decreased. Similar results were found after treated by SH-4-54, inhibitor of STAT5 [31] (Figure 5G). These indicate that IK6 activates downstream STAT5, and HO-1 may be one of the downstream target genes of STAT5.

In brief, all of these data demonstrate that Ikaros protein is mainly synthesized and localized in the cytoplasm due to IK6, and HO-1 is activated by the IK6/STAT5 signaling pathway in BCR-ABL1^+^-IK6 leukemic cells.

## DISCUSSION

Few studies have investigated adult B-ALL, but available data suggest that IK6 might occur at an even higher frequency in adults [13,19,20]. In this study, we found there were several IKZF1 deletion patterns in adult BCR-ABL1^+^ B-ALL patients by using RT-PCR, in which IK6 was the most common type accounting for 47.6%. Our study confirms the statistics reported by Iacobucci et al on the frequency of IK6 (~50%) in adult B-ALL [13,32]. Although lack of the investigation on IK6 expression in BCR-ABL1-negative B-ALL, according to references, the frequency of all abnormal IKZF1 isoforms is around 15% in BCR-ABL1-negative B-ALL [9,15,16], we believe that IK6 is highly expressed in BCR-ABL1-positive B-ALL based on all the facts. In addition, we found that early clinical response was poor in BCR-ABL1-positive B-ALL patients with IK6, and they usually had a higher relapse rate. Thus, IK6 is indeed related to poor prognosis.

The correlation between IK6 and HO-1 has not been reported yet. We studied the expression and role of HO-1 in BCR-ABL1^+^-IK6 leukemic cells to explore their relationship. Results showed that HO-1 was highly expressed in BCR-ABL1^+^-IK6 leukemic cells. And there was a strongly correlation between the expression of IK6 and HO-1. In BCR-ABL1^+^-IK6 leukemic cells, silencing HO-1 resulted in a decreased proliferation and a substantial pro-apoptotic effect, validated by the finding of a significantly enriched subG1 population, an increased proportion of Annexin V-positive cells and an increased expression of Cleaved Caspase3. But it remained almost unchanged in IK6-negative leukemic cells by overexpression of HO-1. This suggests that HO-1 is dependent on IK6 to exert its antiapoptosis and proliferation on cells. It is also need to confirm that HO-1 can promote the proliferation and inhibit apoptosis of BCR-ABL1^+^-IK6 leukemic cells *in vivo*.

We also found that BCR-ABL1^+^-IK6 leukemic cells had a poor response to TKIs. This is consistent with a previous study [13]. These suggest that IK6 expression in BCR-ABL1^+^ B-ALL may contribute to TKI resistance. A major clinical challenge in the treatment of BCR-ABL1**-**positive B-ALL is resistance to TKI [33–36]. Therefore, a number of novel agents are needed to be investigated to overcome drug-resisitance. In our study, silencing HO-1 increased BCR-ABL1^+^-IK6 leukemic cells’ sensitivity to TKIs and led to apoptosis. This phenomenon was also supported by the observation that ZnPPIX substantially augmented the growth-inhibitory effects of imatinib on primary leukemic cells. Moreover, HO-1 inhibitors showed no major effects on viability of normal cells [24–26]. And water-soluble pharmacologic inhibitors of HO-1 has been developed and tested in solid tumors [24,37–40] in the past few years. Therefore, it provides a theoretical basis for HO-1 inhibitors as a novel agent. All of these indicate that HO-1 inhibitors can be considered as a specific for treating TKIs- resistance, especially BCR-ABL1^+^-IK6 patients. But a bigger sample pool of patients is required for further investigations on the verification of HO-1 expression and role in BCR-ABL^+^-IK6 leukemic cells.

**Figure 6:**
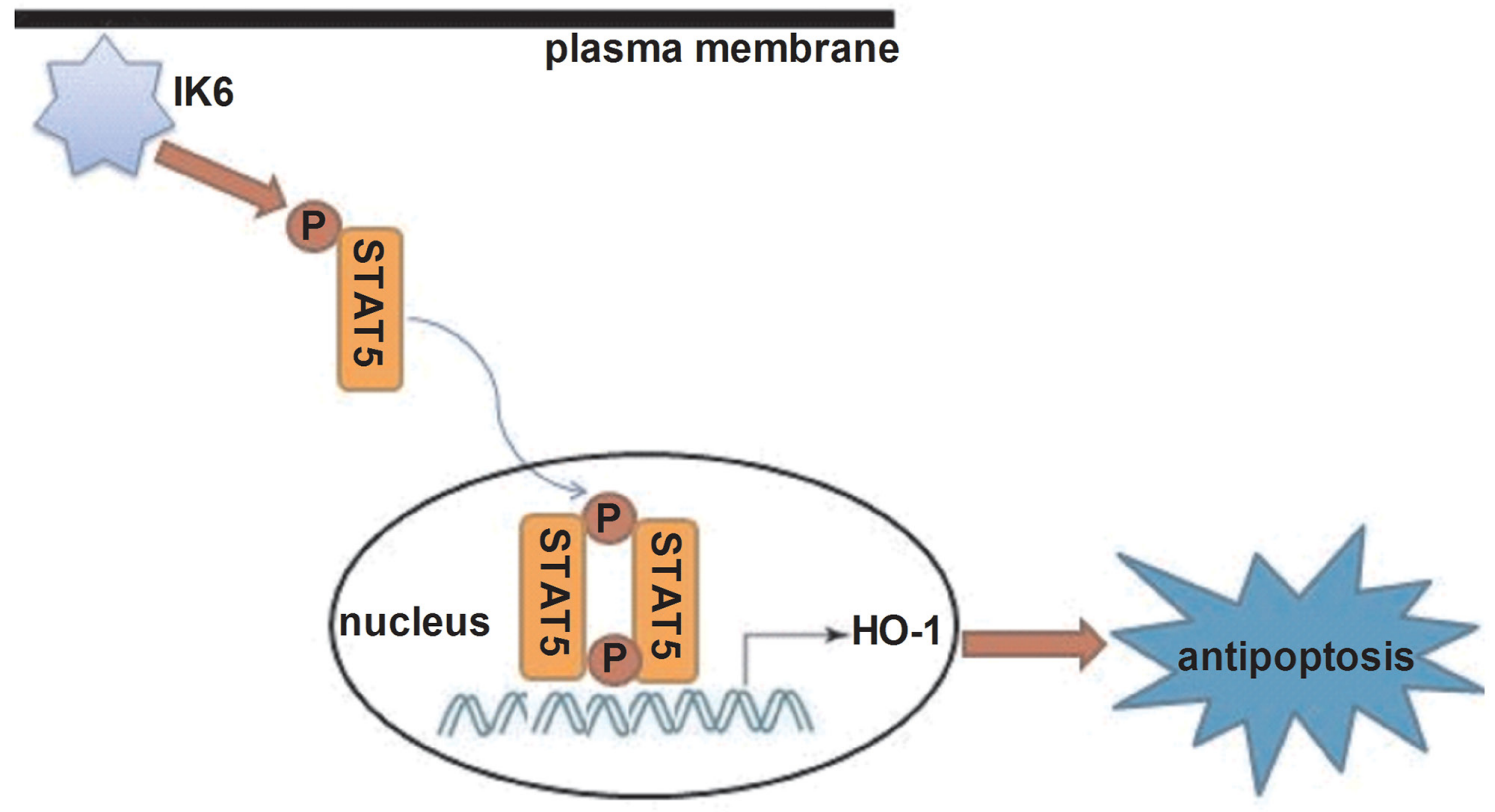
Schematic diagram showing how IK6 activates HO-1

Next, we want to know the pathway of HO-1 involved in antiapoptosis of BCR-ABL1^+^-IK6 leukemic cells. Firstly, experimental results showed that IK6 activated HO-1 and STAT5 signaling in BCR-ABL1^+^-IK6 leukemic cell. But we need to figure out how STAT5 reacts with HO-1. Lentivirus-mediated silencing HO-1 didn’t change the expressions of STAT5 and pSTAT5 in BCR-ABL1**^+^-**IK6 leukemic cells. But the expression of HO-1 was significantly decreased after blocking STAT5 or STAT5 phosphorylation. This shows that IK6 leads to STAT5 activiation, and HO-1 may be one of the downstream target genes of STAT5, which inhibits cell apoptosis [Figure 6]. Our data demonstrate that the signaling pathway (IK6/STAT5/HO-1) suppress the apoptosis of leukemic cells, suggesting that the activation of STAT5 is also closely involved into BCR-ABL1^+^-IK6 leukemic cells’ apoptosis. This accords with STAT5 as a key player involved in apoptosis [41].

It is reported that if IK6 leads to the activation of STAT5, STAT5 inhibitors may offer an alternative strategy for the treatment of BCR-ABL1^+^-IK6 patients [9]. Nevertheless, there have not yet been any convincing demonstrations of STAT5 inhibitors that are both safe and sufficiently specific for human use [42]. However, we found that inhibiting HO-1 reduced the proliferation and induced the apoptosis of BCR-ABL1^+^-IK6 leukemic cells, which enhanced the sensitivity of cells to TKIs. Although HO-1 is in the downstream of STAT5, it can be regarded as one of the targets in treating BCR-ABL1-positive B-ALL with IK6. In conclusion, IK6 results in the activation of STAT5 and HO-1 in the presence of BCR-ABL1. Therefore, we believe that there is a close relationship among IK6, STAT5, HO-1 and BCR-ABL1. However, we still need to figure out how these four genes react with each other.

In summary, inhibition of HO-1 prohibits the proliferation and induces the apoptosis of BCR-ABL1^+^-IK6 leukemic cells, it also increased the sensitivity of cells to TKIs. Therefore, targeting HO-1 can attenuate the negative impact of IK6 in adult BCR-ABL-positive B-ALL.

## MATERIALS AND METHODS

### Patients samples

Bone marrow (BM) samples were obtained from 42 adult BCR-ABL1-positive B-ALL patients at diagnosis whose blast cells were expressed CD34. Patients’ characteristics are shown in Table 2. BM-derived CD34^+^ cells from healthy donors were served as controls. Mononuclear cells (MNC) were isolated by Ficoll-Hypaque (Sigma USA). CD34^+^ progenitor cells (>85% pure) from patients and healthy donors were enriched by using immunomagnetic cell sorting system (MACS) (Miltenyi Biotech, Auburn CA), and frozen in Iscove modified Dulbecco medium (IMDM; Gibco) with 50% fetal bovine serum (FBS; Gibco) and 10% dimethyl sulfoxide (DMSO), then stored in liquid nitrogen, as previously described [43]. This study was approved by the Ethics Committee of Guiyang Medical University in accordance with the Declaration of Helsinki, and all subjects signed consent forms.

### Reagents

Imatinib (STI571) and nilotinib (AMN107) were kindly provided by Novartis Pharma AG (Basel, Switzerland). HO-1 inhibitor ZnPPIX were purchased from Sigma (St. Louis, MO, USA). AZ960 was kindly provided by AstraZeneca R&D (Waltham, MA). SH-4-54 was from Selleck Chemicals (Houston, TX). Primary antibodies for western blot analysis were obtained from Cell Signaling Technology (Beverly, MA), and secondary antibodies were purchased from Beyotime Institute of Biotechnology (Shanghai, China). Primary antibodies used in this study including anti-HO-1, anti- Cleaved Caspase-3, anti-Ikaros, anti-STAT5, anti-phosphorylated-STAT5 (pSTAT5), anti- Actin and anti-LaminB.

### Cell culture

Human BCR-ABL1-positive B-ALL cell lines SupB15 was purchased from the American Type Culture Collection (Manassas, VA). Primary CD34^+^ cells were thawed and set up in suspension culture in IMDM medium with 20% FBS plus 100 U/ml penicillin and 100 mg/ml streptomycin in the presence of recombinant human interleukin-3 (rhIL-3, 20ng/mL, PeproTech, Rocky Hill, NJ), recombinant human interleukin-7 (rhIL-7, 20ng/mL, PeproTech, Rocky Hill, NJ), and recombinant human stem cell factor (rhSCF, 50ng/mL, PeproTech, Rocky Hill, NJ) (3-cytokine mix) [44,45] at 37°C in 5% CO_2_ and humidified atmosphere.

### Reverse-transcribed polymerase chain reaction analysis

Total RNA was extracted by using TRIzol Reagent ((Invitrogen, Carlsbad, CA). RNA was reverse-transcribed into cDNA by using a reverse transcription kit (Takara, Japan). The first round of polymerase chain reaction (PCR) was performed by using specific primer of IKZF1, 5'-CACATAACCTGAGGACCATG-3' (forward) and 5'-AGGGCTTTAGCTCATGTGGA-3'(reverse). Specifically, 2ul cDNA, 12.5uL 2×Taq Master Mix (TianGen Biotech, Beijing, China), 10pmol of each primer, and distilled water were mixed into a final volume of 25uL. The first round condition was 5min at 95°C, followed with 35 cycles at 95°C for 30s, and 57°C for 30s, then 72°C for 90s, finally stopped after 72°C for 7 min. The first round product was used as the template for the second round PCR amplification. The primers 5'-ATGGATGCTGATGAGGGTCAAGAC-3' (foward) and 5'-GATGGCTTGGTCCATCACGTGG-3' (reverse) were designed and synthesized according to the literature [13]. Condition was as follow: 5min at 95°C, followed with 35 cycles at 95°C for 30s, and 62°C for 30s, then 72°C for 30s, finally stopped after 72°C for 7 min. The RNA integrity was confirmed by cDNA amplification of the β-Actin at the level of mRNA.

### Quantitative real-time PCR

Quantitative real-time PCR was performed as described [21,22]. Relative quantification (2^-ΔCT^) method was used for calculating fold changes. β-Actin was used as the internal control. Human HO-1 primers were 5'-ACCCATGACACCAAGGACCAGA-3'(forward) and 5'-GTGTAAGGACCCATCGGAGAAGC-3' (reverse); SOCS2 primers were 5'-GATAAGCGGACAGGTCCAGA-3'(forward) and 5'-AAGAAGGCAAGGCATTCTGA-3' (reverse); SH2B3 primers were 5'-CTTTCCTTATG TGGCAGAGCC-3'(forward) and 5'-GACACCCAGA GACCAAGGAT-3' (reverse); β-actin primers were 5'-GAGACCTTCAACACCCCAGC-3'(forward) and 5'-ATGTCACGCACGATTTCCC-3' (reverse).

### Cell transfection

Recombinant vector were designed and purchased from Biomics Biotechno logies Co., Ltd (Nan tong Jiangsu, China). Cells were transfected according to a previous literature [46] and routine protocols [47]. Briefly, cells were plated onto 12 well plates at 2.5 x 10^5^ cells/well and infected with lentiviral stocks at a multiplicity of infection (MOI) of 10 in the presence of polybrene (10ug/ml), then analyzed by fluorescence microscopy (Olympus, Tokyo, Japan) and western blot 72h post infection. Prior to transfection, cells were cultured in the presence of 3-cytokine mix for 7 days.

### Proliferation assays

After transfection for 72h, cells were washed and continued to culture in IMDM medium with 20% FBS plus 100 U/ml penicillin and 100 mg/ml streptomycin in the absence of 3-cytokine mix. Cell proliferation was analyzed by the Trypan-blue dye exclusion assay. Cells were cultured in 24-well plates at 2×10^4^/ml. The number of viable cells was counted after 24, 48 and 72h by Trypan blue exclusion.

### Apoptosis assays

Cells which were cultured in the absence of 3-cytokine mix were harvested at 72h and washed with phosphate-buffered saline (PBS), then stained with the Annexin-V/7-AAD apoptosis kit (7sea biotech, Shanghai, China) according to manufacturer's instructions. Apoptotic cells were detected by using a FACScan flow cytometry (Becton-Dickinson, Franklin lakes, NJ, USA), and the data were analyzed by using cell fit software.

### Cell cycle analysis

Flow cytometry was used to detect the cell cycle distribution. Cells which were cultured in the absence of 3-cytokine mix were harvested at 72h, and washed with PBS. Cells were then fixed by ice-cold 70% ethanol at 4°C overnight followed by washing with PBS at the second day and staining with 50ug/ml propidium iodide, finally dissolved in 100ug/ml RNase A. All reported values were means of three independent mearsurments with SD.

### Cell viability assays

After transfection, cells were plated in 96-well plates in IMDM medium in the presence of 3-cytokine mix. Imatinib or nilotinib were added into the medium with a gradient concentration. After 48 hours incubation, cell viability was determined by 3-[4,5-Dimethylthiazol-2-yl]-2,5-diphenyltetrazolium bromide (MTT) assay as described [22].

### Sequencing analysis

PCR products were purified with QIAquick PCR purification kit (QIA-GEN) according to the manufacturer's protocol. The purified products reacted with Big Dye Terminator DNA sequencing kit (Applied Biosystems) and sequenced by an ABI 3500 Genetic Analyzer (Applied Biosystems).

### Western blot

Total proteins were extracted by lysing cells in RIPA buffer containing 1 mM Phenylmethanesulfonyl fluoride (Solarbio Science & Technology, Beijing, China). According to manufacturer's instructions, cytoplasmic and nuclear proteins were extracted using the nuclear and cytoplasmic protein extraction kit (Beyotime, Shanghai, China). Western blot was performed as described [21, 22]. We used Lamin B and Actin as control to quantify our nuclear and cytoplasmic fractions. Their optical densities were analyzed with Quantity One software.

### Confocal microscopy

Cells were blocked and then stained with fluorochrome-labeled antibodies. For intracellular Ikaros detection, cells were first permeabilized in detergent-buffered paraformaldehyde and then stained as above using appropriate secondary antibodies. Stained cells were cytospun onto coated slides and mounted with Vectashield containing 4,6 diamidino-2-phenylindole (DAPI). Images were acquired with a FluoView confocal laser scanning microscope (Olympus) and processed with ImageJ.

### Phosphoprotein analysis

Cells were fixed, permeabilized, labeled with pERK, pAKT, pP38, pCREB, pSTAT1, pSTAT3, pSTAT5, or isotype control antibodies, and analyzed by flow cytometry, which was performed as described [48].

### Co-immunoprecipitation binding assay

For co-immunoprecipitation, SupB15 cells in 10 cm dishes were transiently transfected with different plasmids. It was performed as previously described [49,50]. For western blot analysis, anti-Flag and anti-Myc monoclonal antibody (Sigma) were used as primary antibody. To detect endogenous interaction of Ikaros and HO-1, anti-Ikaros and anti-HO-1 antibody were incubated with Dynbeads (Invitrogen), and similar procedures were performed.

### Statistical analysis

All data were analyzed by SPSS ver.19.0 software package (SPSS, Chicago, USA). Statistical significance among groups was determined by analysis of variance (ANOVA) and Student's t-test. The Chi-square test was used to assess the association between IKZF1 status and clinical features. The correlation between the expression of HO-1 and Ikaros was built up by the application of Spearmant. Kaplan–Meier survival curves were plotted and analyzed with the log-rank test. P values less than 0.05 were considered statistically significant. Each experiment was performed in triplicate, data were presented as mean ± standard deviation (SD).

## References

[1] Moorman AV, Harrison CJ, Buck GA, Richards SM, Secker-Walker LM, Martineau M, Vance GH, Cherry AM, Higgins RR, Fielding AK, Foroni L, Paietta E, Tallman MS (2007). Karyotype is an independent prognostic factor in adult acute lymphoblastic leukemia (ALL): analysis of cytogenetic data from patients treated on the Medical Research Council (MRC) UKALLXII/Eastern Cooperative Oncology Group (ECOG) 2993 trial. Blood.

[2] Scherr M, Elder A, Battmer K, Barzan D, Bomken S, Ricke-Hoch M, Schröder A, Venturini L, Blair HJ, Vormoor J, Ottmann O, Ganser A, Pich A (2014). Differential expression of miR-17~92 identifies BCL2 as a therapeutic target in BCR-ABL-positive B-lineage acute lymphoblastic leukemia. Leukemia.

[3] Hoelzer D, Gökbuget N (2000). Recent approaches in acute lymphoblastic leukemia in adults. Crit Rev Oncol Hematol.

[4] Pui C-H, Evans WE (2006). Treatment of acute lymphoblastic leukemia. N Engl J Med.

[5] Heerema NA, Nachman JB, Sather HN, La MK, Hutchinson R, Lange BJ, Bostrom B, Steinherz PG, Gaynon PS, Uckun FM, Children's Cancer Group (2004). Deletion of 7p or monosomy 7 in pediatric acute lymphoblastic leukemia is an adverse prognostic factor: a report from the Children's Cancer Group. Leukemia.

[6] Den Boer ML, van Slegtenhorst M, De Menezes RX, Cheok MH, Buijs-Gladdines JG, Peters ST, Van Zutven LJ, Beverloo HB, Van der Spek PJ, Escherich G, Horstmann MA, Janka-Schaub GE, Kamps WA (2009). A subtype of childhood acute lymphoblastic leukaemia with poor treatment outcome: a genome-wide classification study. Lancet Oncol.

[7] Mullighan CG, Su X, Zhang J, Radtke I, Phillips LA, Miller CB, Ma J, Liu W, Cheng C, Schulman BA, Harvey RC, Chen IM, Clifford RJ (2009). Deletion of IKZF1 and prognosis in acute lymphoblastic leukemia. N Engl J Med.

[8] Martinelli G, Iacobucci I, Storlazzi CT, Vignetti M, Paoloni F, Cilloni D, Soverini S, Vitale A, Chiaretti S, Cimino G, Papayannidis C, Paolini S, Elia L (2009). IKZF1 (Ikaros) deletions in BCR-ABL1-positive acute lymphoblastic leukemia are associated with short disease-free survival and high rate of cumulative incidence of relapse: a GIMEMA AL WP report. J Clin Oncol.

[9] Van der Veer A, Zaliova M, Mottadelli F, De Lorenzo P, Te Kronnie G, Harrison CJ, Cavé H, Trka J, Saha V, Schrappe M, Pieters R, Biondi A, Valsecchi MG (2014). IKZF1 status as a prognostic feature in BCR-ABL1-positive childhood ALL. Blood.

[10] Payne KJ, Dovat S (2011). Ikaros and Tumor Suppression in Acute Lymphoblastic Leukemia. Crit Rev Oncog.

[11] Merkenschlager M (2010). Ikaros in immune receptor signaling, lymphocyte differentiation, and function. FEBS Lett.

[12] Kastner P, Dupuis A, Gaub MP, Herbrecht R, Lutz P, Chan S (2013). Function of Ikaros as a tumor suppressor in B cell acute lymphoblastic leukemia. Am J Blood Res.

[13] Iacobucci I, Lonetti A, Messa F, Cilloni D, Arruga F, Ottaviani E, Paolini S, Papayannidis C, Piccaluga PP, Giannoulia P, Soverini S, Amabile M, Poerio A (2008). Expression of spliced oncogenic Ikaros isoforms in Philadelphia-positive acute lymphoblastic leukemia patients treated with tyrosine kinase inhibitors: implications for a new mechanism of resistance. Blood.

[14] Rebollo A, Schmitt C (2003). Ikaros, Aiolos and Helios: transcription regulators and lymphoid malignancies. Immunol Cell Biol.

[15] Mullighan CG, Miller CB, Radtke I, Phillips LA, Dalton J, Ma J, White D, Hughes TP, Le Beau MM, Pui CH, Relling MV, Shurtleff SA, Downing JR (2008). BCR-ABL1 lymphoblastic leukaemia is characterized by the deletion of Ikaros. Nature.

[16] Iacobucci I, Storlazzi CT, Cilloni D, Lonetti A, Ottaviani E, Soverini S, Astolfi A, Chiaretti S, Vitale A, Messa F, Impera L, Baldazzi C, D’Addabbo P (2009). Identification and molecular characterization of recurrent genomic deletions on 7p12 in the IKZF1 gene in a large cohort of BCR-ABL1-positive acute lymphoblastic leukemia patients: on behalf of Gruppo Italiano Malattie Ematologiche dell’Adulto Acute Leukemia Working Party (GIMEMA AL WP). Blood.

[17] Trageser D, Iacobucci I, Nahar R, Duy C, von Levetzow G, Klemm L, Park E, Schuh W, Gruber T, Herzog S, Kim YM, Hofmann WK, Li A (2009). Pre-B cell receptor-mediated cell cycle arrest in Philadelphia chromosome-positive acute lymphoblastic leukemia requires IKAROS function. J Exp Med.

[18] Iacobucci I, Lonetti A, Cilloni D, Messa F, Ferrari A, Zuntini R, Ferrari S, Ottaviani E, Arruga F, Paolini S, Papayannidis C, Piccaluga PP, Soverini S (2008). Identification of different Ikaros cDNA transcripts in Philadelphia-positive adult acute lymphoblastic leukemia by a high-throughput capillary electrophoresis sizing method. Haematologica.

[19] Mi JQ, Wang X, Yao Y, Lu HJ, Jiang XX, Zhou JF, Wang JH, Jiao B, Shen SH, Tang JY, Gu LJ, Jiang H, Ma LY (2012). Newly diagnosed acute lymphoblastic leukemia in China (II): prognosis related to genetic abnormalities in a series of 1091 cases. Leukemia.

[20] Dupuis A, Gaub MP, Legrain M, Drenou B, Mauvieux L, Lutz P, Herbrecht R, Chan S, Kastner P (2013). Biclonal and biallelic deletions occur in 20% of B-ALL cases with IKZF1 mutations. Leukemia.

[21] Lin X, Fang Q, Chen S, Zhe N, Chai Q, Yu M, Zhang Y, Wang Z, Wang J (2015). Heme oxygenase-1 suppresses the apoptosis of acute myeloid leukemia cells via the JNK/c-JUN signaling pathway. Leuk Res.

[22] Wei S, Wang Y, Chai Q, Fang Q, Zhang Y, Wang J (2014). Potential crosstalk of Ca2+-ROS-dependent mechanism involved in apoptosis of Kasumi-1 cells mediated by heme oxygenase-1 small interfering RNA. Int J Oncol.

[23] Ma D, Fang Q, Wang P, Gao R, Wu W, Lu T, Cao L, Hu X, Wang J (2015). Induction of heme oygenase-1 by Na^+^-H^+^ exchanger 1 protein plays a crucial role in imatinib-resistant chronic myeloid leukemia cells. J Biol Chem.

[24] Mayerhofer M, Florian S, Krauth MT, Aichberger KJ, Bilban M, Marculescu R, Printz D, Fritsch G, Wagner O, Selzer E, Sperr WR, Valent P, Sillaber C (2004). Identification of heme oxygenase-1 as a novel BCR/ABL-dependent survival factor in chronic myeloid leukemia. Cancer Res.

[25] Mayerhofer M, Gleixner KV, Mayerhofer J, Hoermann G, Jaeger E, Aichberger KJ, Ott RG, Greish K, Nakamura H, Derdak S, Samorapoompichit P, Pickl WF, Sexl V (2008). Targeting of heat shock protein 32 (Hsp32)/heme oxygenase-1 (HO-1) inleukemic cells in chronic myeloid leukemia: a novel approach to overcome resistance against imatinib. Blood.

[26] Cerny-Reiterer S, Meyer RA, Herrmann H, Peter B, Gleixner KV, Stefanzl G, Hadzijusufovic E, Pickl WF, Sperr WR, Melo JV, Maeda H, Jäger U, Valent P (2014). Identification of heat shock protein 32 (Hsp32) as a novel target in acute lymphoblastic leukemia. Oncotarget.

[27] Kano G, Morimoto A, Takanashi M, Hibi S, Sugimoto T, Inaba T, Yagi T, Imashuku S (2008). Ikaros dominant negative isoform (Ik6) induces IL-3-independent survival of murine pro-B lymphocytes by activating JAK-STAT and up-regulating Bcl-xl levels. Leuk Lymphoma.

[28] Beer PA, Knapp DJ, Miller PH, Kannan N, Sloma I, Heel K, Babovic S, Bulaeva E, Rabu G, Terry J, Druker BJ, Loriaux MM, Loeb KR (2015). Disruption of IKAROS activity in primitive chronic-phase CML cells mimics myeloid disease progression. Blood.

[29] Yang J, Ikezoe T, Nishioka C, Furihata M, Yokoyama A (2010). AZ960, a novel JAK2 inhibitor, induces growth arrest and apoptosis in adult T-cell leukemia cells. Mol Cancer Ther.

[30] Wilson GS, Tian A, Hebbard L, Duan W, George J, Li X (2013). Tumoricidal effects of the JAK inhibitor Ruxolitinib (INC424) on hepatocellular carcinoma in witro. Cancer Lett.

[31] Xu Y, Wang X, Gao L, Zhu J, Zhang H, Shi H, Woo M, Wu X (2015). Prolactin-stimulated survivin induction is required for beta cell mass expansion during pregnancy in mice. Diabetologia.

[32] Iacobucci I, Iraci N, Messina M, Lonetti A, Chiaretti S, Valli E, Ferrari A, Papayannidis C, Paoloni F, Vitale A, Storlazzi CT, Ottaviani E, Guadagnuolo V (2012). IKAROS deletions dictate a unique gene expression signature in patients with adult B-cell acute lymphoblasticleukemia. PLoS One.

[33] Towatari M, Yanada M, Usui N, Takeuchi J, Sugiura I, Takeuchi M, Yagasaki F, Kawai Y, Miyawaki S, Ohtake S, Jinnai I, Matsuo K, Naoe T (2004). Combination of intensive chemotherapy and imatinib can rapidly induce high-quality complete remission for a majority of patients with newly diagnosed BCR-ABL–positive acute lymphoblastic. Blood.

[34] Yanada M, Takeuchi J, Sugiura I, Akiyama H, Usui N, Yagasaki F, Kobayashi T, Ueda Y, Takeuchi M, Miyawaki S, Maruta A, Emi N, Miyazaki Y (2006). High complete remission rate and promising outcome by combination of imatinib and chemotherapy for newly diagnosed BCR-ABL-positive acute lymphoblastic leukemia: a phase II study by the Japan Adult Leukemia Study Group. J Clin Oncol.

[35] Biondi A, Schrappe M, De Lorenzo P, Castor A, Lucchini G, Gandemer V, Pieters R, Stary J, Escherich G, Campbell M, Li CK, Vora A, Aricò M (2012). Imatinib after induction for treatment of children and adolescents with Philadelphia-chromosome-positive acute lymphoblastic leukaemia (EsPhALL):a randomised, open-label, intergroup study. Lancet Oncol.

[36] Pfeifer H, Lange T, Wystub S, Wassmann B, Maier J, Binckebanck A, Giagounidis A, Stelljes M, Schmalzing M, Dührsen U, Wunderle L, Serve H, Brück P (2012). Prevalence and dynamics of bcr-abl kinase domain mutations during imatinib treatment differ in patients with newly diagnosed and recurrent bcr-abl positive acute lymphoblastic leukemia. Leukemia.

[37] Fang J, Sawa T, Akaike T, Akuta T, Sahoo SK, Khaled G, Hamada A, Maeda H (2003). *In vivo* antitumor activity of pegylated zinc protoporphyrin: targeted inhibition of heme oxygenase in solid tumor. Cancer Res.

[38] Liu ZM, Chen GG, Ng EK, Leung WK, Sung JJ, Chung SC (2004). Upregulation of heme oxygenase-1 and p21 confers resistance to apoptosis in human gastric cancer cells. Oncogene.

[39] Berberat PO, Dambrauskas Z, Gulbinas A, Giese T, Giese N, Künzli B, Autschbach F, Meuer S, Büchler MW, Friess H (2005). Inhibition of heme oxygenase-1 increases responsiveness of pancreatic cancer cells to anticancer treatment. Clin Cancer Res.

[40] Was H, Cichon T, Smolarczyk R, Rudnicka D, Stopa M, Chevalier C, Leger JJ, Lackowska B, Grochot A, Bojkowska K, Ratajska A, Kieda C, Szala S (2006). Overexpression of heme oxygenase-1 in murine melanoma: increased proliferation and viability of tumor cells, decreased survival of mice. Am J Pathol.

[41] Ferbeyre G, Moriggl R (2011). The role of stat5 transcription factors as tumor supressors or oncogenes. Biochim Biophys Acta.

[42] Berger A, Hoelbl-Kovacic A, Bourgeais J, Hoefling L, Warsch W, Grundschober E, Uras IZ, Menzl I, Putz EM, Hoermann G, Schuster C, Fajmann S, Leitner E (2014). PAK-dependent STAT5 serine phosphorylation is required for BCR-ABL-induced leukemogenesis. Leukemia.

[43] Cox CV, Martin HM, Kearns PR, Virgo P, Evely RS, Blair A (2007). Characterization of a progenitor cell population in childhood T-cell acute lymphoblastic leukemia. Blood.

[44] Cox CV, Diamanti P, Evely RS, Kearns PR, Blair A (2009). Expression of CD133 on leukemia-initiating cells in childhood ALL. Blood.

[45] Williams MT, Yousafzai Y, Cox C, Blair A, Carmody R, Sai S, Chapman KE, McAndrew R, Thomas A, Spence A, Gibson B, Graham GJ, Halsey C (2014). Interleukin-15 enhances cellular proliferation and upregulates CNS homing molecules in pre-B acutelymphoblastic leukemia. Blood.

[46] Wang JS, Singh H, Zhang F, Ishizuka T, Deng H, Kemp R, Wolin MS, Hintze TH, Abraham NG, Nasjletti A, Laniado-Schwartzman M (2006). Endothelial dysfunction and hypertension in rats transduced with CYP4A2 adenovirus. Circ Res.

[47] Ma D, Fang Q, Li Y, Wang J, Sun J, Zhang Y, Hu X, Wang P, Zhou S (2014). Crucial role of heme oxygenase-1 in the sensitivity of acute myeloid leukemia cell line Kasumi-1 to ursolic acid. Anticancer Drugs.

[48] Cook AM, Li L, Ho Y, Lin A, Li L, Stein A, Forman S, Perrotti D, Jove R, Bhatia R (2014). Role of altered growth factor receptor-mediated JAK2 signaling in growth and maintenance of human acute myeloid leukemia stem cells. Blood.

[49] Liu Z, Lam N, Thiele CJ (2015). Zinc finger transcription factor CASZ1 interacts with histones, DNA repair proteins and recruits NuRD complex to regulate gene transcription. Oncotarget.

[50] Li S, Wang L, Zhao Q, Liu Y, He L, Xu Q, Sun X, Teng L, Cheng H, Ke Y (2014). SHP2 positively regulates TGFβ1-induced epithelial-mesenchymal transition modulated by its novel interacting protein Hook1. J Biol Chem.

